# Metabolomics on the study of marine organisms

**DOI:** 10.1007/s11306-022-01874-y

**Published:** 2022-03-02

**Authors:** Lina M. Bayona, Nicole J. de Voogd, Young Hae Choi

**Affiliations:** 1 Natural Products Laboratory, Institute of Biology, Leiden University, 2333 BE Leiden, The Netherlands; 2 Naturalis Biodiversity Center, Marine Biodiversity, 2333 CR Leiden, The Netherlands; 3 Institute of Environmental Sciences, Leiden University, 2333 CC Leiden, The Netherlands; 4 College of Pharmacy, Kyung Hee University, 130-701, Seoul, Republic of Korea

**Keywords:** Marine organisms, Metabolomics, Drug discovery, Marine chemical ecology

## Abstract

**Background:**

Marine ecosystems are hosts to a vast array of organisms, being among the most richly biodiverse locations on the planet. The study of these ecosystems is very important, as they are not only a significant source of food for the world but also have, in recent years, become a prolific source of compounds with therapeutic potential. Studies of aspects of marine life have involved diverse fields of marine science, and the use of metabolomics as an experimental approach has increased in recent years. As part of the “omics” technologies, metabolomics has been used to deepen the understanding of interactions between marine organisms and their environment at a metabolic level and to discover new metabolites produced by these organisms.

**Aim of review:**

This review provides an overview of the use of metabolomics in the study of marine organisms. It also explores the use of metabolomics tools common to other fields such as plants and human metabolomics that could potentially contribute to marine organism studies. It deals with the entire process of a metabolomic study, from sample collection considerations, metabolite extraction, analytical techniques, and data analysis. It also includes an overview of recent applications of metabolomics in fields such as marine ecology and drug discovery and future perspectives of its use in the study of marine organisms.

**Key scientific concepts of review:**

The review covers all the steps involved in metabolomic studies of marine organisms including, collection, extraction methods, analytical tools, statistical analysis, and dereplication. It aims to provide insight into all aspects that a newcomer to the field should consider when undertaking marine metabolomics.

## Introduction

Oceans cover most of the earth’s surface, and they are a great source of biodiversity (Cragg & Newman, 2009). Until now, 238,357 species have been accepted, and it is estimated that there are more than 2,000,000 to be discovered (Appeltans et al., 2012; Mora et al., 2011; WoRMS, 2021). The distribution of chemical nutrients and other compounds in a medium that is in continual movement due to waves and currents makes marine ecosystems very dynamic systems (Carr et al., 2003). Moreover, global warming and ocean acidification, a consequence of anthropogenic activities, have modified the environment in the oceans resulting in modifications in the performance of several marine ecosystems (Halpern et al., 2019; Hoegh-Guldberg & Bruno, 2010; Hughes et al., 2003). The exposure of marine organisms to environmental conditions, which can be intrinsic to their ecosystems or induced by anthropogenic activities, results in metabolic changes of these organisms since metabolism is the first to respond to environmental changes experienced by an organism (Viant, 2007).

In this context, a comprehensive analysis of the metabolites produced by marine organisms is crucial to understand the dynamics of marine ecosystems. Such comprehensive analyses can be achieved through metabolomics, an “omics” technology defined as the study of all the metabolites or small molecules present in an organism, cell, or tissue under certain conditions (Bundy et al., 2008; Kim et al., 2011). This kind of untargeted analysis is a powerful tool in the study of marine organisms because it provides a complete overview of the metabolites inside (metabolic fingerprinting) and outside (metabolic footprinting) an organisms to study the metabolic changes caused by genetic, environmental, or biological factors. (Dunn & Ellis, 2005; Goulitquer et al., 2012; Kim et al., 2011). For this reason, metabolomics approaches could provide holistic viewpoint of metabolism, which would be impossible in classical targeted metabolic studies (Ebada et al., 2008). Through unbiased metabolomic analysis, biomarkers for toxicity, diseases, or putative novel molecules with biological activity can be identified without previous knowledge of the specific metabolites or metabolic pathways involved in the studied phenomena (Harvey et al., 2015; Viant, 2007).

One of the most important fields in which metabolomics has been useful is marine chemical ecology which investigates the interactions between marine organisms mediated by chemical compounds (Hay, 2009). This field has been growing in the past 20 years to understand the roles of several chemical compounds produced by different marine organisms (Brown et al., 2019; Hay, 2014; Leão et al., 2012; Mascuch & Kubanek, 2019). Moreover, the use of metabolomics has prompted the identification of compounds involved in ecological interactions such as defense mechanisms against herbivory, allelopathic interactions, and detection of sexual ques (Kuhlisch & Pohnert, 2015). Metabolomics is expected to continue providing valuable information by identifying the metabolites that vary during the interaction between organisms and assigning a putative function to these metabolites.

From another perspective, in natural products, marine organisms have been a prolific source of new compounds. Every year more than 1200 new compound belonging to different families such as alkaloids, terpenoids, peptides, and polyketides are reported from diverse phyla (Blunt et al., 2016, 2017, 2018; Carroll et al., 2019, 2020). In addition, most of the compounds isolated have also shown bioactivity such as anticancer, antibacterial, antifungal and antiretroviral, making marine natural products an interesting source of drug candidates (Gerwick & Moore, 2012; Molinski et al., 2008). Metabolomics has been a promising tool in discovering new active compounds from marine organisms by supporting the selection of putative active compounds early in the pipeline for their isolation and unraveling the mechanism of action of known compounds (Stuart et al., 2020).

Despite the success of the application of metabolomics in the study of marine organisms, its implementation has suffered several practical setbacks that have thwarted the development of its full potential. The collection of significant amounts of sample is often complicated by an extremely low availability, not only due to their natural scarcity but to the difficulties encountered when accessing the most often remote collection sites and/or in their transportation in appropriate conditions to laboratories. Another challenging aspect for the study of marine organisms is inherent to their chemistry since the chemical space occupied for the metabolites isolated from marine organisms is quite broad (Blunt et al., 2018). This chemical space is defined by the study of different physicochemical properties of the metabolites. This represents a challenge for the extraction and detection of all compounds in a single analysis (Kuhlisch & Pohnert, 2015). Moreover, vast amounts of salt and lipids in extracts from sessile organisms such as sponges, corals, and alga require specific sample pretreatment to remove these and obtain extracts that comply with analytical requirements for metabolomic analysis.

Marine organism metabolomics can lead to discovering many bioactive compounds and an increased understanding of the interaction between certain organisms and their environment. In the last few years, several technological advances in existing analytical tools or the development of new related ones have paved the way for exciting applications for metabolomics in this field. This paper reviews all the required steps in the workflow of marine organism metabolomics based on recent studies: sample preparation including extraction, data analysis, and metabolite identification. It also provides examples of the application of metabolomics to a large diversity of organisms and the discussion of scientific and technological advances.

## Sample preparation and extraction: how to maximize the number of intact metabolites in a simple manner

The complexity of marine metabolomics is intrinsic to both the organisms and the environment that they inhabit. Marine organisms establish tight relationships in order to survive the harsh conditions of the marine environment. Thus, when designing an experiment, it is important to bear in mind that this complexity implies that access to marine environments is much harder, requiring more complicated logistics to collect reliable sample sets. Marine ecosystems also differ from terrestrial ones in key features such as salinity, water currents, depths, etc. that must all be considered apart from common ones such as temperature, light, oxygen concentration, for example, among the significant environmental conditions.

The collection of marine organisms is a process with a degree of technical complexity and requires a methodological approach that has to be adjusted according to the organisms that are being studied. Benthic organisms such as corals, sponges, cyanobacteria, algae, mollusk, and tunicates are generally collected by SCUBA divers at depths of 5–40 m; beyond this, in the mesophotic region (40–200 m) and deep-sea, remote operated vehicles (ROVs) and bottom trawls are often used (Hestetun et al., 2019; Jackson et al., 2014; Pham et al., 2019). There are considerable differences between these last two methods: ROVs allow the selective collection of target organisms while bottom trawls remove whole individuals indiscriminately with a negative environmental impact in many cases. When benthic organisms are collected to access their associated microorganisms, several extra steps, including rinsing with sterile water, blending, and fermentation in appropriate culture media, are required to obtain the organism of interest (Li et al., 2012; Said Hassane et al., 2020; Vidal et al., 2020). It is important to note that ethical regulations apply for the study of higher animals such as marine turtles, fish, and marine mammals. Generally, only samples of a specific tissue or biofluid of the animal are allowed to be collected and the process used must ensure the least possible stress to the animal. For instance, non-invasive methods have been used to collect respiratory samples of bottlenose dolphins, and these can be adapted to other cetaceans (Zamuruyev et al., 2016). Due to the difficulties associated with collecting samples from the wild, many culturing systems have been developed, albeit limited to a few organisms. When organisms are aqua- or mariculture, the collection is far simpler, and there are fewer obstacles than those encountered when collecting from the wild and the collection of a wider range of biofluids (tissue, blood and feces) is facilitated.

Once samples are collected, i.e., separated from their natural state, it is generally highly recommended to quench the metabolism immediately, as the collection or harvesting process can per se rapidly produce changes in their metabolism. Many protocols, designed in general for plant or animal tissues, indicate flash freezing the samples using liquid nitrogen (Kim et al., 2010). This, however, is often impossible in the case of marine organisms due to practical constraints related to the installations and available technical equipment at collection locations which are often very distant from a laboratory or similar facilities. Existing alternatives include freezing at – 20 °C using dry ice or adding organic solvents to stop or reduce metabolic reactions. Representative quenching techniques that have been used in marine organism metabolomic studies are listed in Table 1.

**Table 1:** Some of the quenching methods used in marine organism metabolomics studies

Quenching method	Organisms
Centrifugation of the liquid culture media and filtration	Bacteria (Forner et al., 2013; Hou et al., 2012; Viegelmann et al., 2014)
Addition of solvent and freezing at − 20 °C	Cyanobacteria (Luzzatto-Knaan et al., 2017; Winnikoff et al., 2014)
Freeze-drying after harvesting	Cyanobacteria (Kleigrewe et al., 2015)
Addition of solvent and centrifugation	Bacteria (Bose et al., 2015)
Frozen and stored at − 20 °C	Sponge (Ali et al., 2013; Olsen et al., 2016; Reverter et al., 2018) Corals (He et al., 2014a)
Snap frozen using liquid nitrogen	Coral (Farag et al., 2016; Sogin et al., 2014, 2016) Bacteria (Boroujerdi et al., 2009)

Sample treatment of the collected samples involves the extraction of metabolites and the eventual removal of impurities that may be incompatible with the analytical method to be used. The selection of the extraction method includes the choice of a solvent and conditions and is driven by two basic aims: the extraction of as many metabolites as possible and the preservation of their integrity by the elimination or at least reduction of time-consuming steps and manipulation that can lead to the degradation or modifications in the chemical structure of the metabolites. The choice of extraction method also depends highly on the type of sample as well as the organism. Extraction protocols of marine animal blood, feces, and tissue samples are similar to those used for mammalian metabolomics studies (Niemuth et al., 2020; Suzuki et al., 2018) although specific protocols may need to be developed in some cases, e.g., the optimal conditions for the extraction of metabolites from fish feces involved three cycles of rinsing with 2.5% NaCl solution prior to the lyophilization and extraction using acetonitrile/isopropanol/water 3:3:2 (Hano et al., 2018; Wu et al., 2008). On the other hand, whether studying animals or plants, the extraction of benthic organisms follows common protocols used in plant metabolomics and marine natural products studies; organic solvents such as methanol and ethanol or their aqueous mixtures are preferred over pure water or buffers. Naturally, given the chemical diversity of the metabolome of the studied organisms, the solvent of choice will vary according to the predominant or targeted metabolites (Ebada et al., 2008). The efficiency of some extraction systems or related technology and conditions (e.g. temperature and pressure), have been evaluated using algae (Heavisides et al., 2018) and sponges (Bayona et al., 2018) as examples. In these cases, extraction conditions for automatic pressure-assisted extractions systems were evaluated to optimize yields of certain groups of metabolites or the chemical diversity of the extracts. Factors such as temperature, solvent polarity, and the number of cycles were found to influence the diversity of chemical composition of the extracts greatly. However, it is still too early to propose general guidelines for optimum conditions to extract a wider range of marine organism metabolites.

### Clean-up of extracts before analysis

Samples from benthic organisms such as corals and sponges usually contain considerable amounts of seawater. This water contains approximately 3.5% w/w of salt (US Department of Commerce, 2020). Therefore, extracts of these organisms, even those obtained using organic solvents, are likely to contain a high percentage of salt, requiring the inclusion of an extra step for its removal as preparation for most analytical platforms used for their metabolomics study. For example, samples with a moderate to high salt concentration are incompatible with common analytical methods such as LC–MS, HPLC or HPTLC, since salt can affect the resolution of the chromatographic separation and in the case of LC–MS methods that use electrospray ionization (ESI) the presence of salt can damage the ion source. Even in NMR analysis, the high concentration of salt can cause unexpected signal broadening. One of the most common sample pretreatment methods for desalting marine organism extracts is solid-phase extraction with Diaion HP-20 resins, pre-equilibrated with methanol (Houssen & Jaspars, 2012), or C18 and PS-DVB SPE cartridges (Cutignano et al., 2015; Ivanišević et al., 2011). While desalting samples is essential to avoid interferences with sample analysis, it is necessary to validate procedures to ensure that the process does not alter the chemical composition of the extracts.

In addition, some marine organisms contain high amounts of lipids which can cause issues specially for chromatographic analysis since they can attach irreversibly to very commonly used lipophilic stationary phases, causing serious reproducibility problems. Therefore, unless specifically targeted as part of the metabolomic analysis, it is desirable to remove them before the analysis. To this end, Sephadex LH-20 with a mobile phase of methanol and dichloromethane (1:1) or C18 SPE cartridges that retain these highly lipophilic compounds can be used (Houssen & Jaspars, 2012).

## Analytical methods: to detect a variety of intact metabolites in a robust and straightforward manner

Metabolomics studies aim to detect all the metabolites present in an organism under a given set of conditions. Nevertheless, due to the limitations of each analytical platform, it is now accepted that no single method can provide a complete picture of the metabolome. In the case of marine organisms, the limitation is more critical given the vast diversity of the metabolites (Blunt et al., 2018; Carroll et al., 2020). Metabolites of marine sources contain very distinguished chemical moieties such as macro rings (8–10 members), high number of nitrogen and halogen in their structure and stereocenters making them ever more challenging to analyze than those isolated from terrestrial sources (Shang et al., 2018). The structure elucidation of these compounds has been addressed by increasing the performance in various single or hyphenated spectroscopic and chromatographic techniques. This section describes several applications using different metabolomics platforms for marine organism studies.

### Nuclear magnetic resonance spectroscopy: a way of visualizing the overall metabolome

NMR spectroscopy is a widely used analytical technique, mainly for structure elucidation. Since the emergence of metabolomics, NMR, mainly ^1^H NMR, has been one of the most common analytical platforms used for data acquisition together with MS-based methods. However, some other experiments such as J-resolved (Ludwig & Viant, 2010), HSQC (Bingol et al., 2014), and ^13^C NMR (Clendinen et al., 2014) have also been used. Several advantages are associated with NMR-based metabolomics: its high signal robustness, ease of quantification of detected metabolites and most importantly, the structural information provided directly by the spectra themselves. In the past, the evident edge of ^1^H NMR over other analytical platforms for metabolomics resulted in its application to the study of a wide range of organisms such as mussels (Wu & Wang, 2010), shrimps (Schock et al., 2013), sponges (Ali et al., 2013), corals (Sogin et al., 2014) and various fish (Cappello et al., 2018; Melis et al., 2017). In these applications, NMR-based methods showed substantial advantages over other methods, particularly in monitoring the levels of primary metabolites submitted to changes in environmental, biological, or ecological conditions. Overall, NMR allowed the identification of metabolites present in high concentrations such as amino acids, ketone bodies, organic acids, and the energy-related compounds ATP and carbohydrates, which could piece together a metabolic picture of primary metabolism. Although ^1^H NMR is useful to get the profile of the most abundant metabolites, when it comes to detecting and identifying the less abundant metabolites, this technique has some limitations due to its extremely low sensitivity and the congestion of signals in the spectra (Kim et al., 2010; Viant, 2007).

### Liquid chromatography-based methods with mass spectrometer detectors: a closer look at minor metabolites

Liquid chromatography hyphenated to mass spectrometry is the most used technique in marine organism metabolomics. Among its several strong points is the separation provided by the chromatographical step, which also provides additional chemical information as metabolites can be distinguished by their retention time and their m/z value. In LC–MS analysis, hundreds of thousands of metabolites can be detected, including some present at trace levels (Goulitquer et al., 2012). This higher sensitivity is ideal for detecting bioactive and ecologically relevant compounds since, in many cases, these are present in very small quantities (Belarbi et al., 2003).

For example, LC–MS based metabolomics has been widely used to study the metabolites produced by marine microorganisms. The diversity of strains that can be isolated from one sample (marine invertebrate animals or sediments), together with methodological approaches such as OSMAC (Bose et al., 2015; Roullier et al. 2016; Viegelmann et al., 2014), which aim to increase and diversify the metabolic production of the microorganisms by altering the culture condition such as pH, temperature, aeration, and co-culture (Romano et al., 2018), demand a fast and effective method to evaluate their production. Other organisms such as sponges, corals, and algae have been studied for the discovery of new drug candidates, as shown in Table 2. However, molecules cannot be identified based on their molecular mass. Even with high-resolution molecular mass data, reliable fragmentation patterns are needed to confirm the molecular structure. Thus, target compounds must be submitted to tandem MS, i.e., MS^n^ experiments, to generate fragmentation patterns which can then be matched with a reference spectrum, much like a fingerprint. Furthermore, the recent development of the GNPS (Global Natural Products Social network) platform, which uses MS/MS data, has enabled the deduction of structural similarities between metabolites, allowing them to be grouped accordingly (Wang et al., 2016). The detailed procedure and potential of this platform are discussed in the data analysis section.

**Table 2:** Examples of application of metabolomics for the discovery of new bioactive compounds

Organisms	Analytical method	Pattern recognition		Finding	References
		Unsupervised	Supervised		
Bacteria *Vibrio* sp. QWI-06	UHPLC-ESI-Orbitrap-MS	Molecular networking	No	Discovery of vitroprocines A–J active against *Acinetobacter baumannii*	Liaw et al. (2015)
Cyanobacteria *Moorea bouillonii* and *Moorea producens*	HPLC-ESI-Q-TOF–MS	Molecular networking	No	Discovery of three new metabolites columbamides A–C	Kleigrewe et al. (2015)
Cyanobacteria *Moorea producens* JHB	LC-ESI-LTQ-FTICR-MS	Molecular networking	No	Discovery of new compounds of the Jamaicamide and Hectochlorins family	Boudreau et al. (2015)
Bacteria *Salinispora arenícola*	UHPLC-ESI-Q-TOF–MS	PCA	OPLS-DA	The incubation time and salinity of the culture media of *Salinispora arenicola* change the quantity and type of Rifamycins produced	Bose et al. (2015)
Fungi *Aspergillus terreus*	LC-ESI-TOF–MS	PCA	No	Using diverse cultivation media lead to the discovery of the new compound 7-desmethylcitreoviridin	Adpressa and Loesgen (2016)
Bacteria 1000 marine microorganisms	UHPLC-ESI-Q-TOF–MS	Molecular networking and PCoA^a^	No	Increment in the chemical space by using diverse extraction methods and the discovery of two compounds: maridric acids A and B	Floros et al. (2016)
Sponge *Geodia macandrewii* and *Geodia baretti*	UHPLC-ESI-TOF–MS	PCA	OPLS-DA	The differentiation of two sponges of the genera *Geodia* and the isolation of the new compound geodiataurine	Olsen et al. (2016)
Bacteria *Micromonospora* sp. and *Rhodococcus* sp.	UHPLC-ESI-Q-TOF–MS	PCA	No	Discovery of keycin, a new antibiotic from the co-culture of the bacteria	Adnani et al. (2017)
317 marine cyanobacteria and benthic algae	UHPLC-ESI-Q-TOF–MS	Molecular networking and PCoA^a^	No	Differences in the chemical composition of cyanobacteria collected in a different location giving and the discovery of yuvalamine A, a new compound	Luzzatto-Knaan et al. (2017)
Bacteria *Streptomyces* sp. WU20	HPLC-ESI-QQQ-MS	No	PLS	The cultivation of *Streptomyces* sp. WU 20 under nickel stress lead to the discovery of a new compound with antibacterial activity	Shi et al. (2017)
Bacteria 24 *actinobacteria* like strains	HPLC-ESI-IT-MS	HCA	OPLS-DA	24 strains were group depending on their metabolic production. Putative active compounds in quorum sensing assays were selected and dereplicated	Betancur et al. (2017)
Sponge *Spongia officinalis*	HPLC-ESI-Q-TOF–MS	PCA and molecular networking	PLS-DA	Composition of furanoterpenes in *S. officinalis* is changed depending on the geographical location and season. Three new compounds: Furofficin and Spongialactam A and B were isolated	Bauvais et al. (2017)
Fungi 21 isolated fungi	UHPLC-ESI-Q-TOF–MS	Molecular networking	PLS-DA	The co-culture of isolated fungi with phytopathogenic bacteria and fungi trigger the production putative active secondary metabolites. One new putative peptide from the emerimicin family was annotated	Oppong-Danquah et al. (2018)
Alga *Fucus vesiculosus*	UHPLC-ESI-Q-TOF–MS	Molecular networking	No	Seasonal changes in the metabolome the algae were observed and the variation in the concentration of some metabolites was related with changes in the bioactivity of the alga extracts	Heavisides et al. (2018)

^a^Principal coordinate analysis

### Gas chromatography–mass detector hyphenated methods: detailed metabolic analysis for primary metabolites

Although the number of reports of metabolomics studies using GC–MS is notably less than NMR or LC–MS, some examples show the potential of this technique for metabolomics studies of marine organisms. The GC–MS analysis of scallops (*Chlamys farreri*), an important organism in the food industry, was used to evaluate modifications in metabolic pathways related to their survival rates in different preservation conditions. The presence of metabolites indicative of a trade-off between aerobic and anaerobic metabolism detected in live scallops preserved in semi-anhydrous conditions provided essential information for their marketing, since this can affect the final value of the product (Chen et al., 2015). The metabolomes of other organisms, such as starfish and marine polychaeta, have also been investigated. These studies focused on establishing the best extraction and analytical conditions for the optimized detection of metabolites. In both examples, GC–MS analysis provided detailed information of the primary metabolites such as amino acids, lipids, and carbohydrates in the samples (Fernández-Varela et al., 2015; Pereira et al., 2012).

Along with primary metabolites, volatiles have historically been the most frequent targets of GC–MS analysis. In the past, it was suspected that volatiles could play various key physiological roles. In recent decades, they have also come to be considered interesting vectors in the interactions between organisms, given their unique physical characteristics and high diffusion rate. Recently, in situ extraction methods such as headspace extraction and solid-phase microextraction (SPME) have been applied to the analysis of volatile compounds present in marine organisms, such as algae (de Alencar et al., 2017; Jerković et al., 2018) as well as in some marine microorganisms (Barra et al., 2017; Salvatore et al., 2018). The use of in situ methodologies for the extraction and further analysis of this kind of compound can provide additional information in metabolomics studies.

### Other analytical platforms: to complete the metabolome puzzle

The mentioned analytical methods, NMR, LC–MS, and GC–MS, are the most popular in marine organism metabolomics studies. However, despite the increasing analytical range achieved over the past years, there is no single method that can cover the full metabolome of an organism. Therefore, it has become increasingly important to develop novel analytical tools to complement the information obtained by current techniques. In this context, different analytical platforms such as supercritical fluid chromatography (SFC) coupled to mass spectrometry (Shulaev & Isaac, 2018), capillary electrophoresis (CE) coupled to mass spectrometry (Zhang et al., 2017b), infrared spectroscopy (Pilatti et al., 2017), and high-performance thin-layer chromatography (HPTLC) (Bayona et al., 2019) have proved to have the potential to increase the coverage of the chemical space providing a broader view of the metabolome. Nevertheless, most of these minor platforms have not been widely used for marine organism studies, and, at least for the time being, their use is limited as complementary tools to the main analytical platforms solving their specific limitations.

## Data treatment and multivariate data analysis: to extract hidden information from a raw data matrix

The data acquired using any of the described analytical platforms is generally massive and must be profiled in an untargeted manner. This is a key step in any metabolomics study. Data processing involves converting raw spectroscopic and chromatographic data to a numerical matrix that can be used for statistical or multivariate data analysis. Each analytical method requires different steps in order to obtain the numerical matrix. In general, the preprocessing of NMR data is somewhat simpler than that required for other analytical tools and consists of phase correction, baseline adjustment, shift adjustment, binning [e.g. dynamic adaptative binning (Anderson et al., 2011), fuzzy warping (Wu et al., 2006) and peak alignment using a genetic algorithm (Forshed et al., 2003)], and normalization (Izquierdo-García et al., 2011). For LC–MS data, several software programs are available for data processing, some of which are open access, such as MZMine 2 (Pluskal et al., 2010), OpenMS (Röst et al., 2016), XCMS (Smith et al., 2006), and MS-DIAL (Tsugawa et al., 2015). Although each software uses its own algorithms with different steps, they share the goal of creating a matrix that contains the intensity of all molecular features in each sample and removes possible technical variations. Alignment of the molecular features in both retention time and mass values is seen as an essential part of LC–MS data processing, and variability in retention times, mass values, and isotopic patterns must all be considered.

Regardless of the analytical methods used for metabolomics analyses, a huge number of variables will always be generated. This number can range from several hundred to tens of thousands of variables. Therefore, the first important step in data mining is the reduction of data dimensionality to extract hidden information from the raw data matrix. Multivariate data analysis (MVDA) is usually divided into two categories: unsupervised and supervised methods. For unsupervised MVDA, many approaches have been developed to reduce the dimensionality of the data, out of which principal component analysis (PCA) is currently the most commonly applied in marine metabolomics. PCA aims to collect most of the variance present in a data set using new orthogonal variables, known as principal components (Worley & Powers, 2013). There are a number of examples of marine organism studies in which PCA was successfully employed: the metabolic discrimination of three species of crabs (*Callinectes sapidus*, *Eriphia verrucosa* and *Cancer pagurus*) (Zotti et al., 2016), the differentiation of bacterial strains that produce new compounds (Macintyre et al., 2014), and the detection of metabolic changes caused by exposure to steroids in mussels (Cappello et al., 2017). Similar to unsupervised methods, there are numerous methods that can be applied for supervised approaches in many biological systems in marine organisms. Typical examples of the applications of various unsupervised methods are shown in Tables 2 and 3.

**Table 3:** Examples of application of metabolomics to environmental and biological studies of marine organisms

Organisms	Analytical method	Pattern recognition		Finding	References
		Unsupervised	Supervised		
Coral *Pocillopora damicornis*	UHPLC-ESI-Q-TOF–MS and GC-EI-TOF–MS	No	OPLS-DA	Exposure to different conditions of temperature and pCO_2_ shift metabolic pathways including carbohydrate metabolism, cell structural maintenance, defense mechanisms among others	Sogin et al. (2016)
Coral *Sarcophyton* spp., *Lobophytum pauciflorum*, and *Sinularia polydactyla*	UHPLC-ESI-LCQ-MS and ^1^H NMR	PCA	OPLS	Coral growth in the wild exhibit higher levels of cembranoids, the most common group of diterpenes reported for soft corals, while corals growing in an aquarium have a higher content of oxylipins	Farag et al. (2016)
Alga *Fucus vesiculosus* and *Fucus serratus*	GC-EI-TOF–MS	MDS^a^	dbRDA^b^	For two algae belonging to *Fucus* genus changes in the surface chemistry during different season was observed. Compounds such as, saccharides, hydroxy acids and fatty acids were found to be up regulated during the summer and environmental conditions such light were found to be related with these metabolic changes	Rickert et al. (2016)
Bacteria *Persicivirga* (Nonlabens) *mediterranea* TC4 and TC7 *Pseudoalteromonas lipolytica* TC8 and *Shewanella* sp. TC11	UHPLC − ESI-Q-TOF–MS	PCA and molecular networking	PLS-DA	Different culture conditions are reflected in the metabolome of the four bacteria studied. Compounds of the family of hydroxylated ornithine lipids, diamine lipids and glycine lipids are putative biomarkers	Favre et al. (2017)
Alga *Ulva mutabilis*	GC-EI-TOF–MS and UHPLC-ESI-TOF–MS	PCoA^c^	CAP^d^	The exo-metabolome and development of *Ulva mutabilis* is changed by the presences of symbionts	Alsufyani et al. (2017)
Clams *Ruditapes philippinarum*	^1^H NMR	No	PLS-DA	The analysis of *R. philippinarum* gills after been exposed to hypoxia showed changes in the concentration of some amino acids and energy related metabolites	Zhang et al. (2017c)
Sea snail *Haliotis diversicolor*	^1^H NMR	PCA	OPLS-DA	The exposure of *H. diversicolor* to organotin contaminant cause changes in the energy metabolism, osmotic balance oxidative stress. Moreover, the metabolic response is different depending on the sex and the tissue analyzed	Lu et al. (2017)
Mussels *Mytilus galloprovincialis*	^1^H NMR	PCA	No	The exposure of *M. galloprovincialis* to drospirenone has no effect in their sexual development but disrupt energy, amino acids, and glycerophospholipid metabolism	Cappello et al. (2017)
Macroalga *Asparagopsis taxiformis* Coral *Astroides calycularis*	UHPLC–ESI-QTOF-MS	PCA	No	The interaction between a coral and an invasive alga revealed no changes in the metabolome of the coral while the metabolome of the alga was changed when in contact with the coral	Greff et al. (2017)
Fish *Salvelinus alpinus*	^1^H NMR	PCA	PLS-DA	The metabolic changes caused by a test diet were reflected in the metabolome of the plasma, liver and muscle. Based on this, improvements in the diet were proposed	Cheng et al. (2017)
Sponge *Haliclona mucosa* and *Haliclona fulva*	UHPLC-ESI-Q-TOF	PCA	No	Differences in the metabolome between the two species were observed. Additionally, a decrease in the diversity of the metabolome during the period between April and May was observed and variation due to the location was detected over a 200 km ratio in the Mediterranean Sea	Reverter et al. (2018)
Fish *Thunnus thynnus*	^1^H NMR	PCA	No	Study of the liver tissue of bluefin tuna show differences in the metabolic changes according to the gender, caused by the accumulation of environmental contaminant. Energy-related metabolites, amino acids and lipids were identified as the most affected metabolites	Cappello et al. (2018)
Dinoflagellate *Karenia brevis* Diatom *Asterionellopsis glacialis*	^1^H NMR	PCA	PLS and OPLS-DA	It was found that the allelopathic effect of *K. brevis* was variable and, in some conditions, it could also be no-allelopathic or even stimulate the growth of *A. glacialis*. Signal related with aromatic and polyunsaturated alkenes were increased in the allelopathic samples but also in the one with no allelopathy or stimulatory activity, this suggest specific compounds and not a family of compounds are responsible for this effect	Poulin et al. (2018b)

^a^Multi-dimensional scaling

^b^distance-based redundancy analysis

^c^Principal coordinate analysis

^d^Canonical analysis of principle coordinates

## Databases: both for the dereplication of known metabolites and for a repository for future identification

The final aim of any metabolomics study is the identification of metabolites corresponding to the signals selected from the MVDA. However, the process between selecting a signal and identifying the corresponding metabolites is arduous, time-consuming, and usually considered the bottleneck in metabolomics studies. In this sense, data sets from marine organisms can be particularly demanding due to the chemical diversity of their metabolomes. The first step in the annotation of the metabolites is to dereplicate the compounds using available databases (Blunt et al., 2012). There are two types of databases used in metabolomics studies: general chemical entities and spectral data libraries. The general chemical databases such as PubChem (PubChem, NIH), ChemSpider (ChemSpider, RCS), the CAS registry through SciFinder (SciFinder, CAS), Dictionary of Natural Products (DNP 28.2, CRC Press), Super Natural II (Banerjee et al., 2015), AntiBase (AntiBase, Wiley), and the Natural Products Atlas (NP Atlas) (van Santen et al., 2019) are available online. These databases provide information on nomenclature, molecular formula and mass, source and physicochemical properties of the compounds. Databases such as PubChem and ChemSpider, are non-specialized and cover all kinds of chemical entities regardless of their origin. On the other hand, databases like Super Natural II, Dictionary of Natural Products, NP Atlas, and AntiBase focus on compounds isolated from specific natural sources such as plants, animals, or microorganisms.

The uniqueness of the chemical features of marine natural products, especially those from marine invertebrates, has resulted in the development of specialized databases. MarinLit is the most important database in the marine natural products field, containing more than 27,500 compounds reported from marine sources (MarinLit, RSC). It offers a wide range of options among its search criteria, including spectroscopic data such as UV maximum, NMR chemical shifts, and the exact mass. In the past years, another two open-sourced databases that focus on marine natural products have become available. MarinChem3D is a database that includes the tridimensional structure of over 30,000 marine natural products and some of their physicochemical propierties (MarinChem3D.). The most recently released is the Comprehensive Marine Natural Products Database (CMNPD), based on the annual marine natural products reviews, and includes all marine natural products reported from 1960 until 2018 (Lyu et al., 2020). Besides the chemical structure of 31,000 MNP, this database also provides some of their physicochemical and pharmacokinetic properties, biological activity, taxonomy, and geographical distribution of their source organisms. Information on the geographical distribution of the source organism can be particularly interesting for ecological studies. Other databases such as AntiBase and NP Atlas are useful for the dereplication of compounds and compound families isolated from marine microorganisms, a field that is growing due to the evidence of the potential of marine microorganisms as sources of new and bioactive compounds.

The second type of databases focus on spectral information and are very useful for dereplication in the field of metabolomics. These databases are usually specialized in one analytical technique. NMR databases such as SpecInfo-Consortium Member-NMR, IR and MS (SpecInfo-Consortium Member-NMR, IR and MS, Wiley), and Chenomx contain information on the ^1^H NMR and/or ^13^C NMR spectra of several thousand compounds. Particularly for ^1^H NMR spectra, complete databases are required for full identification, as signals in a specific region of the spectra can be assigned to different compounds or kinds of compounds and detailed information on specific compounds is very helpful. In the case of GC–MS data, the widespread use of electronic impact (EI) as an ionization method generates highly reproducible fragmentation patterns that allow reliable identification using databases. In contrast, LC–MS analysis has produced less reproducible fragmentation data. In the past, analyses focused on the mass of adduct ions because of their soft-ionization modes. However, recently the necessity of tandem mass information (MS/MS and MS^n^) for identification has become evident. Databases such as NIST 20 (NIST 20), MassBank (Horai et al., 2010), and METLIN (Guijas et al., 2018) have available MS/MS spectra that can be used to perform dereplication of compounds, including different ionization methods such as EI, ESI, and MALDI coupled to various mass analyzers. These databases enable matching putative compounds using both their molecular mass and the MS/MS spectra. Additional search features, such as fragment losses in the MS/MS spectra can help to find analogs of unknown compounds.

As with any other metabolomics studies, marine metabolomic studies greatly rely on the information available in databases to annotate compounds. As mentioned, the specialized databases MarinLit and AntiBase are particularly useful for the identification of secondary metabolites. However, the use of generic databases should not be dismissed, as several metabolomics studies of marine organisms have revealed changes in primary metabolites that may not be available in specialized libraries. Mass spectrometry databases are continually updated with additions of new information/molecules, making them a powerful tool for the dereplication of compounds, even if differences in experimental parameters and different instrument might greatly influence the mass spectra. In the case of NMR metabolomics, it is important to note that there is a need for the inclusion of more NMR spectroscopic data of marine natural products in the databases to improve the annotation rate of compounds. Moreover, the in silico predictions of NMR spectra, already available in software such as ACD/Labs, could benefit from the addition of more experimental data into the databases. Fortunately, with the increase in public spectroscopic data that is now required in metabolomics studies and the inclusion of this information in the databases, an increase in the annotation of compounds in marine metabolomics studies can be expected.

## Statistical total correlation (STOCSY), small molecule accurate recognition technology (SMART) and molecular networking (MN): in silico identification of metabolites from complex mixtures

A reasonable approach for identification is to use currently available databases as shown in the previous section. However, the limited number of entities in databases (e.g., NMR and MS spectra) was an incentive for the development of in silico identification strategies using both NMR and MS based methods. The different nature of the features obtained from NMR and MS data has led to the development of diverse approaches.

**Fig. 1:**
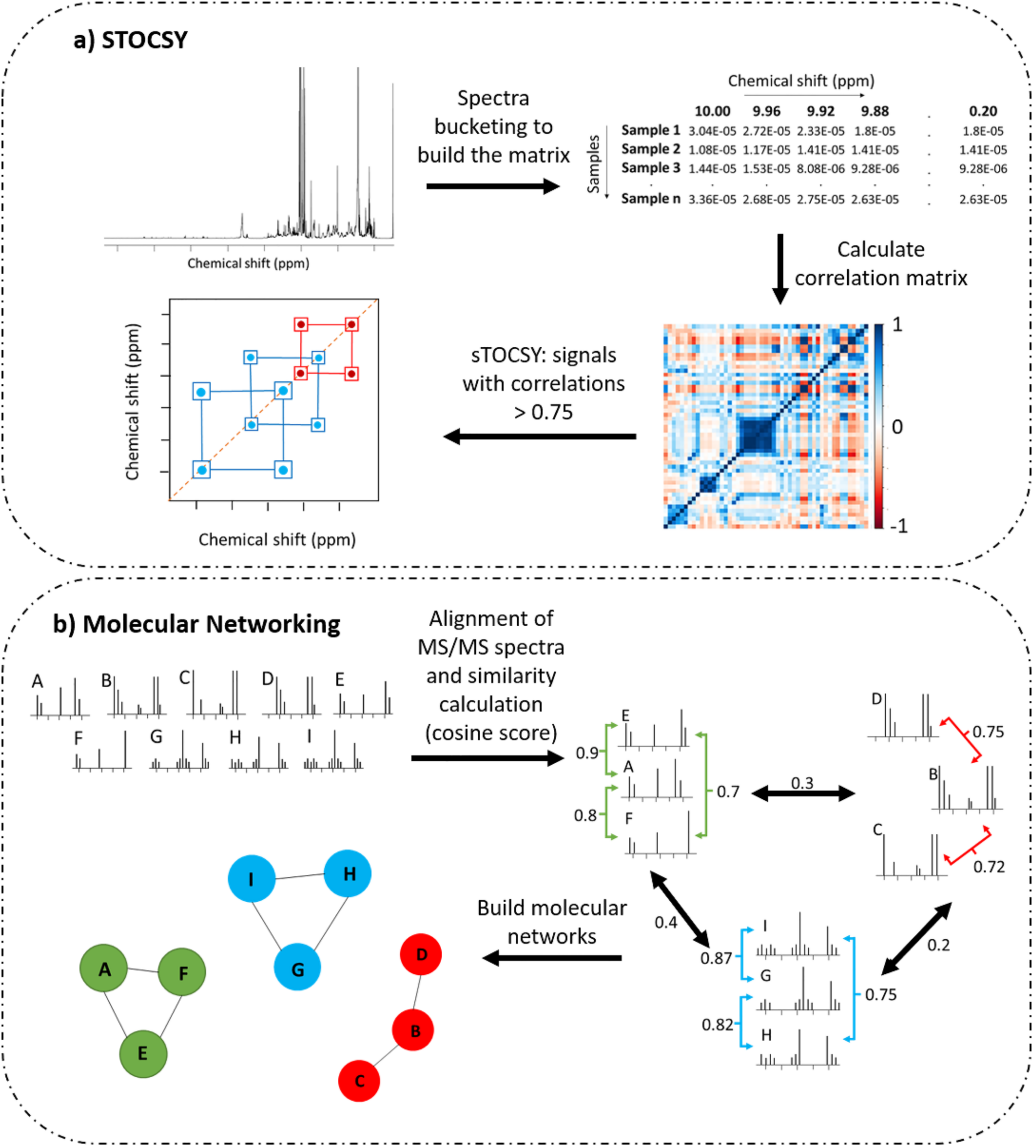
Comparison of the workflows of **a** STOCSY (statistical total correlation spectroscopy) and **b** Molecular networking. **a** The correlation matrix shows the correlation values. Those with correlation values above 0.75 are plotted in the spectra. In red are the correlations that are also present in TOCSY spectra as a result of chemical bonds, and in blue are the correlations between signals that are not chemically bonded. **b** Shows the alignment of MS/MS spectra and calculation of the similarity cosine score, features with scores above 0.7 are connected adapted from Watrous et al. (2012)

The use of computational tools has increased the amount of information that can be extracted from NMR spectra and consequently the identification of compounds in a mixture. One of the computational tools used in some marine organism metabolomic studies is statistical total correlation spectroscopy (STOCSY). This method uses the correlation between the intensity of the signals in the ^1^H NMR spectra to distinguish which signals belong to the same compound or family of compounds (Fig. 1a), facilitating the identification of some molecules (Lindon & Nicholson, 2008). STOCSY was used to identify compounds such as alanine, trigonelline, threonine and lactate, present in coral extracts in a study that aimed to distinguish the chemical profiles of reef-building corals (Sogin et al., 2014). It was also used to identify the changes in the metabolic profile of breast cancer cells treated with candidate anticancer marine natural products (Bayet-Robert et al., 2010). It was possible to determine that when treated with ascididemin, a compound isolated from the marine tunicate *Didemnum* sp., the tumor cells accumulated unusual amounts of gluconic acid.

Other computational tools based on NMR data, combine HSQC spectra with deep convolutional neural networks in a new platform called Small Molecule Accurate Recognition Technology (SMART) for the dereplication of natural products (Zhang et al., 2017a). This platform enables rapid dereplication of molecules into a family of compounds. In combination with metabolomics platforms based on MS data, such as molecular networking, it can lead to the fast and efficient discovery of new molecules. This was the case for viqueamide C, a new cyclopeptide isolated from marine cyanobacteria *Rivularia* sp. and *Moorea producens* (Gerwick, 2017). This molecule was highlighted by both molecular networking and SMART approaches as a new compound from the family of viqueamides, leading to its isolation and confirmation of its structure.

Molecular networking (MN) is a metabolomics workflow based on MS-data sets, which has emerged in the last few years (Yang et al., 2013). When it was first introduced, it was used as a dereplication methodology, but this was later extended to the visualization of complex connections between all metabolites, which could reveal metabolic correlations in biosynthesis. The construction of molecular networks is based on the analysis of MS/MS spectra of compounds, assuming that molecules with similar chemical structures should display similar fragmentation patterns. The basic idea of MN data calculation is to give cosine scores to MS/MS spectral similarity: the closer the score is to 1 the higher the similarity between spectra, with a threshold usually set at 0.7 (Fig. 1b) (Quinn et al., 2017). Recently, a new workflow called feature-based molecular networking was developed (Nothias et al., 2020). This network has some advantages compared to traditional Molecular Networking as isomers can be distinguished based on differences in retention times and the quantification of the features in the samples is more accurate as it uses peak area instead of ion intensity.

The power of MN lies in grouping the metabolites with structural similarities. This has proved to be very useful for metabolomics and particularly in marine organism metabolomics, such as the study of three marine cyanobacteria strains *Trichodesmium erythraeum*, *Okeania* sp., and *Oscillatoria* sp. that allowed the identification of the aplysiatoxins family from clusters obtained from the molecular networks (Ding et al., 2018). The *T. erythraeum* strains collected in different locations displayed different nodes in the cluster related to aplysiatoxins depending on the location where the samples were collected. In contrast, for *Okeania* sp. and *Oscillatoria* sp., this cluster was not changed due to the location. In addition, other clusters observed in the molecular networks were shared between the three cyanobacteria indicating that similar environmental conditions can trigger the biosynthesis of similar metabolites among these cyanobacteria. Ecologically, this investigation is an example of the strong influence of environmental factors on the metabolic production of these cyanobacteria. From a chemical perspective, it provided an example of the possibility of identifying compounds within clusters using the structural similarity to known compounds present in this same cluster (Yang et al., 2013).

Even with existing databases, the chance of success in identifying metabolites would still be low, mainly due to the limitation of data entities, which require time-consuming measurements. To circumvent this limitation, in silico dereplication tools, especially in silico MS fragmentation, appeared a few years ago as a solution to the lack of experimental information. Following the proposal of the Wolfender group to use in silico dereplication together with molecular networks (Allard et al., 2016), many researchers have followed suit and developed platforms with this purpose. Platforms such as network annotation propagator NAP (da Silva et al., 2018), MS2LDA (van der Hooft et al., 2016), and MolNEtEnhancer (Ernst et al., 2019) have all been implemented in the GNPS webpage, to be used together with molecular networks for the identification of molecules or families of molecules.

## Metabolomics applications to marine organisms: biological, ecological, pharmaceutical, and physiological studies

### Marine natural products and discovery of new bioactive compounds

The structural novelty of marine natural products shows great potential, as they can provide a multitude of leads for the development of novel drugs. There are, however, a few critical limitations for the isolation of natural products from marine organisms: the structural complexity of their metabolites, their extremely low concentration, and the limited amount of source materials. Considering that these organisms cannot be easily obtained simply by cultivating or farming, therefore techniques to work with small amounts of extracts need to be optimized. Therefore, any successful project for drug discovery from marine sources must adopt an approach that can speed up the identification of bioactive molecules in these relatively adverse circumstances.

In recent years, and similarly to other fields of life sciences, metabolomics has been increasingly applied to the study of marine natural products as a new holistic tool for metabolic mining. Metabolomics platforms have increased the chances of discovering chemicals with either novel chemical structures or bioactivities. For example, phallusialides A–E, a group of alkaloids, were identified from the marine bacteria *Micromonospora* sp. using a metabolomics approach (Zhang et al., 2019). The LC–MS metabolomic study showed that among 72 bacterial strains belonging to this genus, one was clearly distinguished based on its distinctive chemical profile. This was observed in the PCA analysis where this strain appeared completely separate from all other samples; the separation was attributed mainly to one compound with an *m/z* value of 403.16 (phallusialide A). After this initial identification, molecular networking analysis allowed the selection of other analogs of this compound, out of which four additional compounds were later isolated and identified (phallusialides B–E).

It is well known that the production of active compounds by organisms can be triggered or inhibited by environmental or growth conditions in which they develop, whether in natural environments or in controlled laboratory conditions. In the case of laboratory grown organisms, metabolic profiling is useful to select the conditions that result in the production of more active or unique compounds, an optimization that is essential for successful aqua- and mariculture. Thus, metabolomics together with OSMAC (One Strain Many Compound) has been used to identify the optimal culture conditions for different microorganisms, thus increasing the chemical diversity of the extracts obtained. One example of this is a study on fungi associated to the algae *Fucus vesiculosus* (Fan et al., 2019). Using a molecular networking approach, the differences in the chemical composition of several fungi strains affected by culture conditions (solid vs liquid), culture media (potato dextrose, modified Wickerham, sucrose yeast and Czapek medium) were identified; more importantly, it was possible to distinguish between bioactive, toxic and non-active extracts. Thus, the implementation of a metabolomics approach allowed the selection of the fungi strain and optimal culture conditions needed to obtain the highest yield of anticancer compounds.

Another important aspect to consider in the discovery of active metabolites in living organisms is the effect of environmental conditions on their production. The environment is particularly important for sessile organisms such as corals and sponges because they rely heavily on the conditions of the location where they settle after the larval period. Recently, an LC–MS-based metabolomics study showed that the Caribbean soft coral *Erythropodium caribaeorum* produced different diterpenoids of the erythrolides family in three different locations of the Caribbean (Molina et al., 2020). Interestingly, specimens from one of the locations (Santa Marta) showed higher chemical diversity, with at least ten different erythrolides, while specimens from Islas del Rosario only showed two major compounds, erythrolides A and B, which incidentally proved to be the most active against human cancer cell lines. This study proved the importance of the effect of environmental conditions on the production of bioactive compounds.

Besides their use as sources of prodrugs, marine microorganisms have recently proved to be an interesting alternative in the search for compounds to combat phytopathogens, increasing the potential application in agriculture. A metabolomics approach could enable the investigation of the marine bacteria and fungi combinations that increase the production of metabolites that can then be used in the protection of plants against phytopathogens. As expected, the co-culture of fungi and bacteria from marine environments and phytopathogens was found to trigger the production of compounds that could be bioactive. For example, the use of LC–MS based metabolomics and a molecular networking workflow allowed the identification of five metabolites from the co-culture of eight fungi isolated from marine environments and several common phytopathogens (Oppong-Danquah et al., 2020). Among the identified compounds, cephalochromin showed activity against *Xanthomonas campestris* and *Phytophthora infestans* in the same order of magnitude as the positive control. Marine bacteria have proved to be a prolific source of active compounds against phytopathogens. For example, a *Streptomyces* strain isolated from marine algae was found to produce active compounds against the rice pathogen *Burkholderia* spp. (Betancur et al., 2020). Using an NMR-based metabolomics approach, it was possible to establish relationships between some NMR signals and the bioactivity, leading to the identification of two phenylethylene amides that were responsible for the activity. To extend their potential use in agriculture, marine microorganisms have been investigated for their biocontrolling capacity against phytopathogens by inoculating the microorganism in the plant or soil (Lara-Capistran et al., 2020; Ortega-Morales et al., 2009; Radovanović et al., 2018). In this kind of marine research, metabolomics allowed not only the identification of the active compounds but provided information allowing a deeper understanding of the relationship between the microorganism of marine origin with the phytopathogen and the plant (Allwood et al., 2008).

In general, the procedure for the discovery of bioactive molecules using metabolomics in marine organisms consists of metabolic profiling, bioactivity testing of samples, the detection of correlations between features generated by these using MVDA, the identification of signals responsible for positive correlations, and the annotation of the metabolites corresponding to the signal/s. In Table 2, a number of applications of metabolomics to the discovery of new bioactive molecules from various marine organisms are listed.

### Biological and ecological applications

The discovery of new molecules from marine organisms focuses mainly on sedentary organisms such as sponges, corals, algae, and microorganisms. Biological or ecological studies pose a greater variety of research questions and include higher animals such as fish, mussels, and turtles. The diversity of organisms in these studies results in a great heterogenicity in the type of samples. The preanalytical and analytical protocols used for sponges, corals, and other sessile organisms can be similar to the ones used in general drug discovery studies. However, for other organisms such as fish, mussels, and marine mammals, samples from organs, tissues, and biofluids require preanalytical treatment similar to those used in human metabolomics studies.

With continued population growth, nature is continuously challenged by human activities, and ecosystems’ survival has been increasingly imperiled. This is aggravated by the lack of knowledge about the real implications of the effects caused by human activities on these ecosystems. Currently, many human activities produce massive amounts of residues such as pesticides, antibiotics, hormones, and heavy metals that often find their way to the waterways and ocean, polluting them. In the past few years, a number of studies involving the effect of synthetic chemical residues on the metabolome of a wide range of marine organisms have been actively carried out. Felline et al. studied the effect of Roundup®, a glyphosate-based herbicide, on the metabolome of the algae *Fucus virsoide* (Felline et al., 2019). This herbicide has been widely used, and its residues have been found in fresh water and the marine environment. Changes in the metabolome of *F. virsoide* were registered even when exposed to 0.5 mg/ml, the lowest concentration used. These changes were primarily related to the shikimate pathway, with an increase in the accumulation of shikimate and a decrease in the content of aromatic amino acids which could ultimately affect synthesis of secondary metabolites and proteins.

In addition, the increasing presence of pharmaceuticals in marine environments has become evident in the past few years (Mezzelani et al., 2018). This could cause serious issues in marine organisms affecting them in unexpected ways, despite not being the main target of the drugs. Using LC–MS based metabolomics, Bonnefille and co-workers have studied the effect of diclofenac, a pharmaceutical drug found in one of the highest concentrations in the environment, on marine mussels (*Mytilus galloprovincialis*) (Bonnefille et al., 2018). Changes in the metabolism of tyrosine and tryptophan were detected, particularly an increase in the levels of normetanephrine, serotonin and 5-hydroxyindoleacetic acid, all of which have been reported to be involved in osmoregulation in other marine organisms. In mussels, the interruption of this process could lead to the disruption of homeostasis and changes in the excretion of nitrogenous waste compounds. Moreover, catecholamines and serotonin are known to be involved in the reproduction of mussels, particularly in the development of gametes and sexual maturation.

Some marine organisms were investigated to biomonitor the presence of pollutants such as heavy metals in marine environments. Although mussels have been widely used for this purpose, alternative organisms such as clams have also been considered due to their wide geographical distribution. Ji et al. used NMR based metabolomics together with antioxidant enzymatic analysis to select the most suitable of two pedigrees (white or zebra) of clams *Ruditapes philippinarum* for biomonitoring the presence of heavy metals (Ji et al., 2015). In general, differences in the metabolome of those two classes of clams were found to be related with amino acids such as valine, isoleucine, leucine and histidine and energy-related metabolites such as ATP and succinate. Results also revealed differences in the response of white and zebra clams to their exposure to zinc and cadmium: white clams accumulated more Cd in their digestive tissues and elevated levels of valine, isoleucine, leucine, arginine, glutamate, acetoacetate and succinate and a decrease in hypotaurine and ATP. Thus, these clams proved to be more sensitive to heavy metals exposure and consequently more useful in their biomonitoring in marine environments. This study showed the adequacy of the implementation of a metabolomics approach to detect relevant differences in the response to environmental stressors of otherwise very similar organisms that could be unnoticed if studied with other methods.

The number of studies that use a metabolomic approach to explain physiological processes of marine organisms such as mating, predator defense, and growth development, all of which are likely mediated by chemical compounds, have been growing gradually, especially those related to sessile animals. Reverter and co-workers studied the seasonal and reproductive stage variation of secondary metabolites in the marine sponge *Aplysina cavernicola* (Reverter et al., 2016). They found that the production of secondary metabolites increased with the higher temperatures experienced during the summer months while no correlation was associated to the reproductive stage. A further analysis of the bioactivity of the sponge extracts against four pathogens showed that it was related to the concentration of dienone. The concentration of dienone was one of the compounds found to be increased in summer; this increase in concentration could be an associated protective response as higher summer temperatures can favor the proliferation of pathogens. Thus, the possibility of correlating large datasets provided by comprehensive metabolomic profiling allowed the discovery of possible defense mechanisms derived from the identification of differential chemical features.

A deeper understanding of defense mechanisms can have multiple applications. Among these, the recognition of self and foreign cells and the differentiation between microorganisms that are part of the own microbiome from those that are a potential threat is very interesting. Although some of these mechanisms are known in humans, they can frequently differ significantly in the case of marine animals. Quinn et al. studied the changes in the metabolome of corals caused by the interaction with other corals and algae, the latter being one of the main space competitors of corals in reefs (Quinn et al., 2016). They found that the interaction of with other organisms increased the chemical diversity of their holobiont. More importantly, they concluded that when corals suffer damage associated with the competition with algae, the ratio between PAF (palette activating factor) and Lyso-PAF was affected, with an increase in the amount of PAF. This compound is known to be a pro-inflammatory signal in the human immune system, suggesting that immune responses could be conserved through all metazoans.

In addition to defending themselves, marine organisms can also avoid being attacked by hiding from their predators. For this, some organisms rely on chemical cues to notice the presence of a possible threat. This is the case of mud crab (*Panopeus hebstii*) that uses compounds releases in the urine of its predator, the blue crab (*Callenectus sadipus*), to detect their presence (Poulin et al., 2018a). Using ^1^H NMR-based metabolomic Poulin et al. observed chemical divergence in the urine of blue crabs depending on their diet. It was found that blue crabs fed with a mud crab diet showed an increase in the levels of several compounds in their urine. Particularly two compounds, trigonelline, and homarine, were found to be responsible reduce the feeding behavior of mud crabs, which indicates that these compounds are waterborne cues that alert of the presence of blue crabs to mud crabs.

Lastly, one of the central and most substantial issues in marine biology is taxonomical classification. Many of the traditionally used taxonomic characteristics are not useful for the classification of marine organisms and the available knowledge on genetic markers is still scarce. Classical characterization has, in many cases, resulted in misclassifications of many species, generating a constant creation and reclassification of species. The use of metabolomics in this field could provide an additional taxonomical marker or at least a holistic overview of the metabolome, which is unlikely to be achieved with conventional methods. For example, metabolic fingerprinting was implemented to distinguish two morphotypes of the zoanthid, *Parazoanthus axinellae*, collected in different locations of the Mediterranean Sea. This resulted in the detection of taxonomic marker type metabolites: ecdysteroids, zoanthoxanthins, and parazoanthines (Cachet et al., 2015). Parazoanthines were found to be present only in the “slender” morphotype during the whole year but not in the other morphotype. This result endorsed the revision of the classification of *Parazoanthus axinellae*.

A summary of more applications of metabolomics to biological and environmental sciences is shown in Table 3.

### Connecting the metabolome: an insight into the role of metabolites

To reach a deeper understanding of the role of some metabolites in marine organisms determining the localization of their synthesis and storage is just as important as discovering which factors favor or prevent their production. Nowadays, this is possible thanks to direct infusion MS techniques such as matrix-assisted laser desorption/ionization (MALDI) imaging, direct analysis in real time (DART) and desorption electrospray ionization (DESI) all of which not only provide ecologically relevant information about where compounds are produced and/or stored but can offer the possibility of analyzing samples without extracting them from their matrix (Esquenazi et al., 2009; Parrot et al., 2018). In marine organisms, MALDI-TOF imaging has been used to detect metabolites such as viridamides, jamaicamides and curacin in filaments of marine cyanobacteria consortium, assigning the production of certain metabolites to specific cyanobacteria (Esquenazi et al., 2008). In the same study, the distribution of metabolites within the sponge *Dysidea herbacea* cells was shown to be non-homogenous. This application is an example of the invaluable contribution of MALDI-TOF imaging, providing information on the spatial distribution of secondary metabolites in the tissues of marine organisms and the identity of low molecular mass molecules within a potentially interfering matrix.

The application of a mass imaging-metabolomics approaches to study ecological interactions studies has also been a powerful approach for studying ecological interactions involving, for example, surface-related phenomena and spatial localization of metabolites. A recent report describes the combination of LC–MS based metabolomics with mass imaging to study the chemical defense mechanism of eelgrass against microbial foulers (Papazian et al., 2019). In this case, an untargeted metabolomics revealed different concentrations of a group of fatty acids and phenolic compounds in a leaf surface extract and the whole leaf of the marine plant *Zostera marina*. These compounds were targeted in DESI-MS imaging analysis, showing that some fatty acids were more abundant on the surface of the leaf while phenolic compounds were more abundant in the inner tissues of the leaf. This information allowed the proposal of a defense mechanism of *Zostera marina* against microbial foulers.

Interactions between host and pathogens can be unsynchronous process and sometimes studies performed in bulk liquid media will not reflect the changes in the metabolome happening during the infection process. Schlyer et al. studied the infection of the bloom-forming algae *Emiliania huxleyi* by its specific virus (EhV) by combing a plaque assay with mass imaging spectrometry (Schleyer et al., 2019). The study of the viral infection using MALDI-MS and Flow-Probe-MS led to the identification of change in 24 lipid compounds, out of which 19 have not been described previously in this infection model. Most of the undescribed compounds contain odd chain fatty acids (C15:0) that were mostly incorporated into the cytoplasmic membrane and lipid droplets. They proposed the shift toward odd chain metabolism as a strategy of the virus to take control over the host metabolism.

Another aspect that must be considered is the relatively low representation of microbial biodiversity from marine environments among the information in the available libraries of marine compounds; every year, more than 1200 new marine organism-sourced compounds are reported (Carroll et al., 2020), but this number is insignificant compared with the biodiversity estimations. Therefore, creating marine microorganism libraries is a very important task and has attracted the attention of the scientific community not just from a theoretical viewpoint related to biodiversity per se but because of the interest in the bioactive compounds that can be produced by these microorganisms. However, in the search for microorganism strains that can produce new compounds, the redundancy in the microbial isolates conspires against the construction of useful libraries. Metabolomics based on MALDI-TOF MS proved to be a useful tool for the depuration of microbial libraries. Using a new online platform called IDBac it is possible to select the colonies which differ most and have greater chemically diversity (Costa et al., 2019). This platform requires two MS spectra per sample, one ranging from 1.9 to 2.1 kDa to record the protein region and one between 50 and 2700 Da for the specialized metabolite range. The first experiment is used to create a pseudo phylogenetical tree, while the natural products spectra are used to create metabolite associated networks. With the pseudo phylogenetical tree and the metabolite networks, it is possible to observe metabolic overlapping between colonies. This platform was designed to assist users in the selection of bacterial colonies for the creation of libraries.

## Summary and perspectives

Although knowledge of marine organisms and their ecosystems has been rapidly increasing over the past decades, with more species and thousands of new molecules reported every year, the scale of unexplored organisms is thought to be astounding. Moreover, even with the developments in technologies such as SCUBA diving, ROV’s (Remotely operated vehicles), and research submersibles, which allow a study of the environment that could not even be imagined in the past, the study of marine organisms remains an arduous task. The application of metabolomics in marine organism studies provides a new approach for the discovery of compounds that can be used for the benefit of humans in different ways. Perhaps even more importantly, it has increased the understanding of the function that all these metabolites have within the producing organism as well as the interactions of these organisms with their environment.

Previous studies have suggested associated of microorganisms to be the real synthesizers of many of the secondary metabolites isolated from marine invertebrates (Gerwick & Moore, 2012; König et al., 2006). This has prompted the study of marine associated microorganisms, where new active novel compounds can be discovered using metabolomics as a criterion to select strains for the study. The main focus of marine chemical ecology and environmental metabolomics is the study of interactions of marine organisms with their environment and with other organisms. There is particular interest in the study of the effect of modifications in environmental conditions resulting from human activities. Within this field, metabolomics-based analyses could be expected to lead to increased understanding of the interactions between some organisms and their symbionts, or biosynthetic pathways of compounds and how these are transferred between organisms (He et al., 2014b; Mascuch & Kubanek, 2019; Miyazawa & Noguchi, 2001). In this case, metabolomics studies, together with studies on the biosynthetic pathways can lead to the discovery of how and why compounds are produced.

The ultimate goal of metabolomics, that is, acquiring a picture of the whole metabolome of an organism, is still a chimera, as none of the existing analytical platforms can detect all metabolites in a single analysis. The addition of new analytical tools, such as CE-MS, SFC-MS, HPTLC, and IR-based metabolomics, is a step in this direction, but even then, the use of more analytical platforms does not necessarily guarantee more useful information. Rather, it is increasingly clear that the solution to this problem may come via the combination of available metabolomics tools in a way that can provide the most useful information about the particular organism under study using bioinformatics tools.

Identification of the metabolites of interest continues to be the major restriction in metabolomics studies. Although dereplication can be performed in the initial steps, on many occasions, the compounds cannot be detected within the mixture and have to be isolated for their identification. In the isolation process, the limited amount of sample is always an obstacle, and this has been circumvented in some cases by the implementation of microscale isolation protocols for low amounts of crude extracts. Additionally, other dereplication tools such as in silico MS/MS comparison can be useful for the identification of compounds, as reference compounds needed for identity confirmation are generally very difficult, if not impossible to acquire. The use of these strategies can facilitate metabolomics studies and speed up the process of discovering new compounds and thereby reveal the function of these compounds in the organisms.

Until now studies have focused on the metabolites present inside the organisms, ignoring those that are released into the ocean. To achieve a deeper understanding of the interactions occurring in marine organisms, it is crucial to study the metabolites that are released into the water, as it is highly likely that these metabolites are possible mediators in these interactions. However, the study of exuded metabolites represents a major experimental challenge, as they are rapidly diluted in the ocean making their analysis very difficult. As a first approach, studies could be performed in the laboratory, in a controlled environment that reproduces the conditions in the field. In addition, water filtering systems could allow the concentration of the metabolites using methods such as solid-phase extraction. The use of this kind of experiment enables the collection of metabolites in a sufficient quantity to perform further metabolomics analyses. These kinds of studies will become an indispensable tool to understand the transfer of metabolites between organisms, providing valuable information for fields such as aquaculture, marine chemical ecology, and environmental metabolomics.
